# *In vivo* lineage tracing reveals *Axin2*-expressing, long-lived cortical thymic epithelial progenitors in the postnatal thymus

**DOI:** 10.1371/journal.pone.0184582

**Published:** 2017-09-08

**Authors:** Si Hui Tan, Roel Nusse

**Affiliations:** 1 Program in Cancer Biology, School of Medicine, Stanford University, Stanford, California, United States of America; 2 Department of Developmental Biology, Howard Hughes Medical Institute (HHMI), Institute for Stem Cell Biology and Regenerative Medicine, School of Medicine, Stanford University, Stanford, California, United States of America; University of São Paulo, BRAZIL

## Abstract

In the thymus, cortical and medullary thymic epithelial cells (TECs) are instrumental for generating a repertoire of functional T cells. Hence, there has been much interest in the ontogeny of TECs. While medullary TEC (mTEC) and bipotent progenitors have been identified, the existence of a cortical TEC (cTEC) progenitor remains ambiguous. In this study, we used lineage tracing based on a target gene of the Wnt pathway, *Axin2*. We found that Axin2 initially labels cells in both the cortical and medullary compartments. Using *Axin2*-CreERT2 mice to track the fate of labelled cells, we identified long-lived cortical TEC progenitors that give rise to expanding clones and contribute to homeostasis in postnatal thymus. In contrast, no clonal expansion was found in the medullary or in the K5K8-double positive compartments. The identification of cTEC progenitors and their regulation by Wnt signaling have important implications for our understanding of thymus physiology during homeostasis and TEC-related disorders.

## Introduction

In jawed vertebrates, the thymus is a critical site where immunity is established [1]. During development, immature thymocytes home to the thymus and begin the intricate process of expansion and selection, eventually emerging as functional T lymphocytes that perform cell-mediated immunity. T lymphocyte maturation process is orchestrated by stromal thymic epithelial cells (TECs), which guide migrating thymocytes to undergo negative and positive selection [1]. Defects in TECs can lead to pathologies like immune deficiency or autoimmune diseases like Myasthenia gravis [2], underscoring the importance of TECs in ensuring proper development of the immune system. While the identities of the embryonic TEC stem and progenitors have been established [3], comparable knowledge about postnatal TECs is just starting to emerge.

The thymus is organized into two compartments—the outer cortex and the inner medulla. The TECs that reside within each compartment are functionally different, and are termed cortical TECs (cTECs) and medullary TECs (mTECs) respectively. In the current TEC ontogeny model, differentiated cTECs (marked by cytokeratin 8, K8, expression) and mTECs (marked by cytokeratin 5, K5, expression) originate from a bipotent progenitor. This bipotent progenitor, thought to express both K5 and K8 [1,4], has been identified by transplantation of embryonic TECs marked by MTS24 [5–8] or EpCAM [2,9], as well as lineage tracing [3,10]. Recently, a bipotent progenitor in the adult thymus has also been identified via transplantation with aggregated fetal thymus cells in the kidney capsule [11,12]. A mTEC progenitor capable of initiating and maintaining medullary islets from embryogenesis through adulthood is also known through lineage tracing studies [10,13–16]. In contrast, evidence for a cTEC progenitor in adult animals has been scarce—the two oft-cited evidences are (i) a single cTEC-only clone observed in embryonic lineage tracing [10] and (ii) ontogenetic analysis of putative embryonic cTEC progenitors based on marker expression, without validation of the cells’ behavior *in vivo* or *in vitro* [17].

Studies of Wnt signaling in the thymus have mainly focused on its role in regulating the various stages of thymopoiesis [18]. However, Wnt signaling also plays a critical role in TEC biology since FoxN1, the determinant of the entire TEC lineage, is a Wnt target gene [19]. A handful of studies have demonstrated Wnt signaling as an important regulator of TECs during development [20], homeostasis [21], and regeneration [22]. To study the TECs that engage in Wnt signaling, we labeled and mapped the fates of cells expressing *Axin2*, a Wnt target gene, [23] using the Axin2-CreERT2 (AXCT2) mouse [24]. Through *in vivo* lineage tracing, we map *Axin2*-expressing postnatal cTEC progenitors that contribute to thymic epithelial homeostasis.

## Materials and methods

### Animals

Axin2CreERT2 mice were previously described [24]. mTmG reporter mice [25] were obtained from The Jackson Laboratory (Bar Harbor, ME). All experiments were approved by the Stanford University Animal Care and Use Committee (protocols # 8937and #16646) and performed according to NIH guidelines.

### Lineage tracing studies

Tamoxifen (Sigma-Aldrich, St. Louis, MO) was dissolved in 90% corn oil/10% ethanol, and filtered through a 0.2um membrane. P16 mice were injected with 15mg tamoxifen/25g body weight or 0.1mg/25g intraperitoneally. Mice were sacrificed 48 hours, 1, 2, 3, 6, and 12 months after tamoxifen administration by carbon dioxide asphyxiation. Corn oil controls were injected with a comparable amount of corn oil/ethanol mixture adjusted to their weight.

### Sample processing

For Axin2LacZ samples, thymuses collected were fixed in 4% paraformaldehyde (wt/vol) for 2 hours at 4°C, then incubated in 30% sucrose at 4°C overnight, and embedded in OCT. For AXCT2;mTmG samples, thymuses collected were immediately fixed in 4% paraformaldehyde (wt/vol) overnight at 4°C. The next day, they were incubated in 30% sucrose/PBS (wt/vol) at 4°C overnight, then embedded in OCT.

### X-gal staining

Frozen samples were sectioned and kept at -20°C until ready for staining. The thymuses were stained with 1mg/mL X-gal solution overnight in the dark at 37°C. The next day, the thymuses were washed in PBS, and counterstained in Nuclear Fast Red (Vector Laboratories Inc, Burlingame, CA), then dehydrated through alcohol series, cleared in Orange Terpene, then mounted with EcoMount (BioCare Medical, LLC, Concord, CA).

### Immunostaining

Frozen samples were sectioned at 6μm using with the cryostat (Leica Microsystems Inc, Buffalo Grove, IL). To remove endogenous mTomato signal, antigen retrieval was performed by heating the slides in Citra solution (BioGenex, Fremont, CA). The slides were blocked in 5% Normal Donkey Serum (Jackson ImmunoResearch Laboratories, Inc, West Grove, PA) in 5% PBST (phosphate buffered saline with 0.02% Tween-20) at room temperature for 1 hour, then stained with primary antibody diluted in blocking buffer overnight at 4°C. The next day, the slides were washed in PBS 3 x 10min, incubated in secondary antibody in blocking buffer for an hour at room temperature, and washed in PBS 3 x 10min before mounting on ProLong Gold DAPI (Life Technologies). The primary antibodies used were rabbit anti-Keratin 5 (1:1000, Abcam, Cambridge, MA), rat anti-Keratin 8 (1:250, Developmental Studies Hybridoma Bank, Iowa) and chicken anti- GFP (1:1000, Abcam). Anti-Rabbit-Cy3, anti-Chicken-AlexaFluor488, and Alexa Fluor 647-Strepravadin (all from Jackson ImmunoResearch) were used for the secondary antibody for immunofluorescent staining.

### Microscopy and imaging

Fluorescent immunostaining images were taken as z-stacks on the Leica SP8 Confocal Microscope (Leica Microsystems). The z-stacks were then processed using Maximal Projection on the LAS AF software. The images for cell counting and X-gal-stained samples were taken on Zeiss Axio Imager 2 microscope.

### Labeling frequency

The initial labeling frequency was determined by using tiled images of whole cryosections of P16-P18-traced AXCT2;mTmG thymuses (stained for GFP, K5 and K8). The total number of cells was given by DAPI staining, and the number of labeled cells was given by the number of nuclei that were bound by mGFP staining. (n = 4 mice).

### Quantification of cluster size and type

Immunostaining against GFP, K5 and K8 were performed as described above. Two to three sections were sampled per thymus, with the sections being at least 30μm apart from each other. In each section, all GFP+ cells are imaged. They were then identified for colocalization with K5 and/or K8, and the number of cells was counted by identifying the number of nuclei completely bound by mGFP. The numbers then tabulated for further analyses.

The p values of significance were derived by applying two-tailed unpaired Student’s t-test on specific pairs of cluster types. Statistics are presented in the format of average±SEM, unless otherwise stated.

## Results

### Postnatal cortical and medullary thymic epithelial cells express Axin2

**Fig 1:**
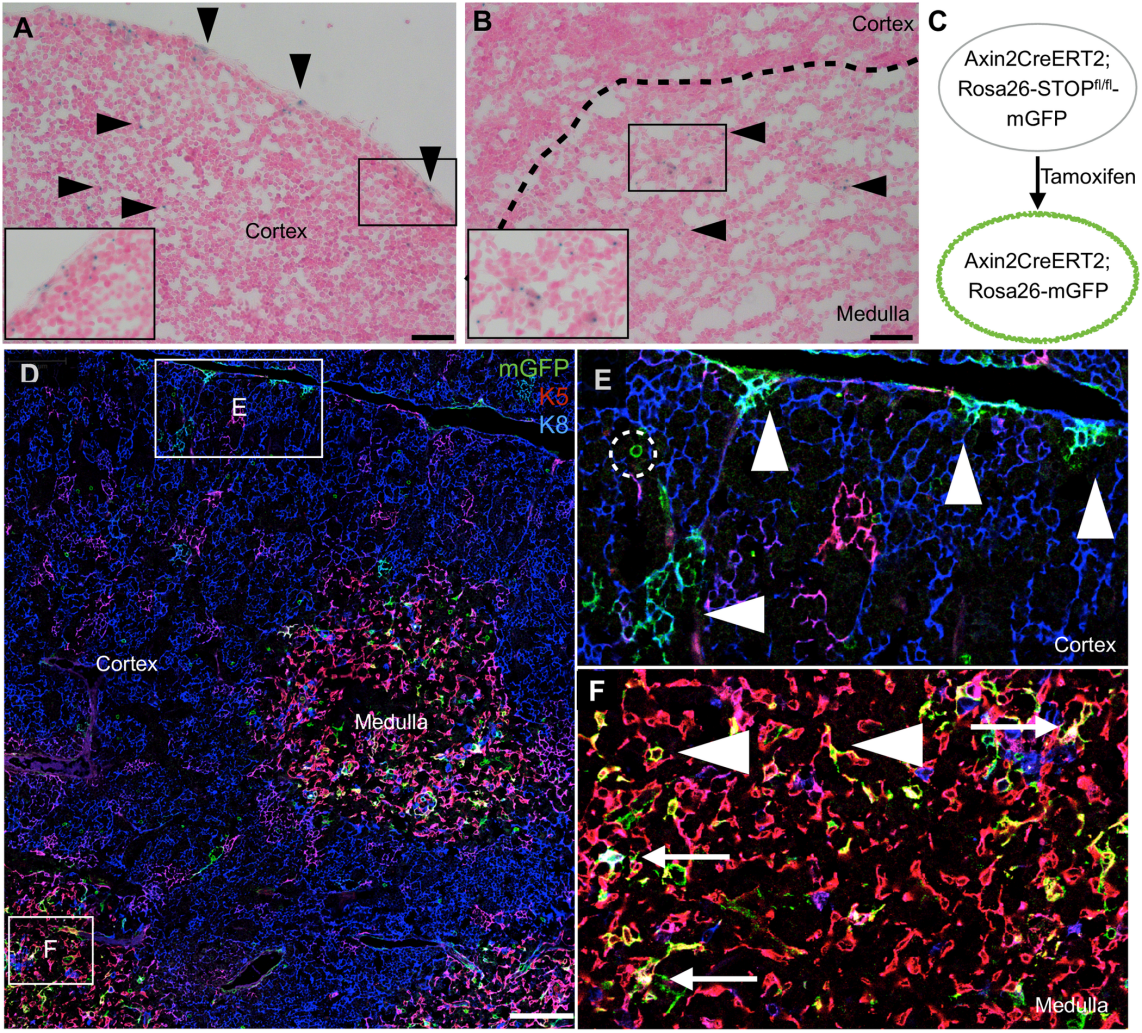
Postnatal thymic epithelium contains *Axin2*-expressing cells in both cortex and medulla. **(A to B)** P16 Axin2-lacZ thymus stained with X-gal. Arrowheads point to X-gal stained cells, and insets are magnified views of the boxed regions. **(C to F)** AXCT2;mTmG thymus traced with a high dose of tamoxifen (15mg tamoxifen/25g body weight) from P16-P18 and stained for K5 and K8 expression. Boxed regions in (D) are magnified in (E) and (F). (E) Cortical region showing clusters of AXCT2-labeled K8+ cTECs after two days (arrowhead). Some cells with circular morphology are observed, and are absent from the thymus after one month. (F) Medullary region showing a more dispersed pattern of AXCT2-labeled cells, which express K5 only (arrowhead), or both K5 and K8 (arrow). Scale bars for (A) and (B) represent 40μm, and 100μm for (D).

We surveyed the expression of the Wnt target gene, Axin2 in P16 Axin2-LacZ reporter mice [23,26] and found discrete LacZ-expressing cells in both the cortex (Fig 1A) and medulla (Fig 1B). In particular, we consistently observed LacZ-expressing cells near the edge of the cortex relatively frequently (Fig 1A). We then examined AXCT2;mTmG reporter mice [24,25] during a short-term trace, from P16 to P18 (Fig 1C–1F). These mice were pulsed with a high dose of tamoxifen (15mg/25g body weight) to label *Axin2*-expressing cells at high frequency. The labeled cells had the stellate morphology typical of TECs, and we proceeded to colocalize *Axin2*-expressing cells with TEC markers. The large majority of the mGFP-labeled cells were either cTECs (marked by K8 expression)(Fig 1D and 1E, arrowheads) or mTECs (marked by K5 expression, arrowhead)(Fig 1D and 1F). A small subset of the mGFP-labeled cells is double positive (DP) for K8 and K5, a pattern suggested to mark putative bipotent progenitors (Fig 1F, arrow) [4], though not always at the cortico-medullary junction, where the bipotent progenitors are thought to reside [4]. We concluded that subsets of postnatal K8- and/or K5- marked TECs express *Axin2*.

In addition, we observed rare mGFP-labeled cells with a circular morphology, distinct from TECs’ stellate morphology (Fig 1E, dotted circle). As these labeled cells were absent from the thymus after one month of tracing, we focused on *Axin2*-expressing TECs that persisted in the thymic epithelium over the long term.

### AXCT2 labels multiple TEC subpopulations

**Fig 2:**
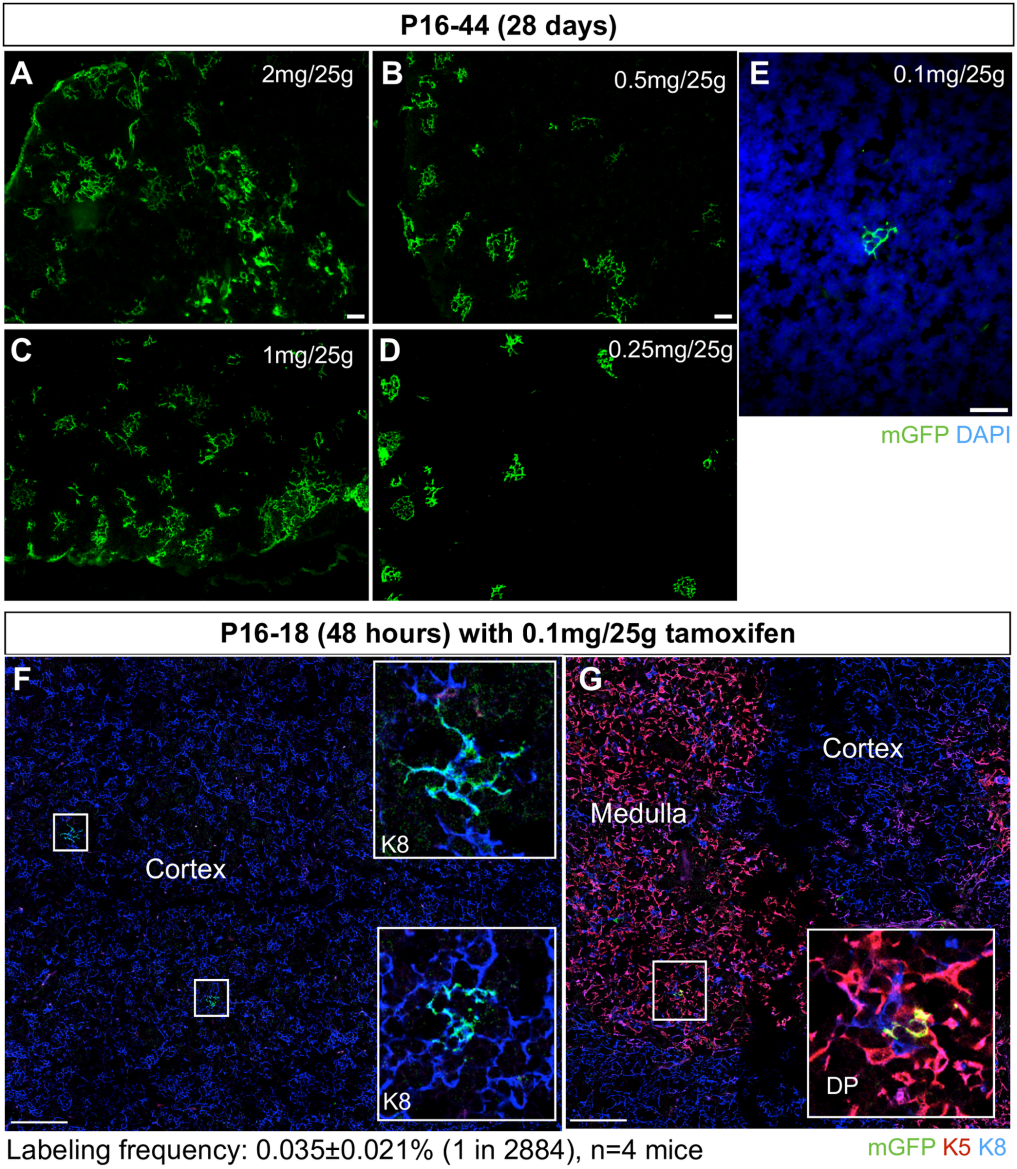
Titration of tamoxifen dosage to achieve sparse labelling. **(A to E)** Dosages of 2mg tamoxifen/25g body weight, 1mg/25g, 0.5mg/25g, 0.25mg/25g and 0.1mg/25g were titrated and the labeled clusters were examined 28 days post tamoxifen administration (P16-P44 trace). **(F and G)** Sparse labeling of TECs is observed in the cortex (F, K8 positive) and medulla (G, K5 K8 double positive) after 48 hours of inducing labeling. Insets are magnified views of the boxed regions showing labeled cells. Labeling frequency is sampled by imaging the entire thymus, and calculating the proportion of the total number of cells (DAPI-labeled) that are AXCT2-labeled (mGFP-labeled). Scale bars in (A) to (E) represent 100μm, and 20μm and (F) and (G).

When genetically labeled and traced over time, stem cells and long-lived progenitors give rise to persistent clones that increase in size, whereas non-stem/progenitor cells will generate transient or non-expanding clones. We applied this reasoning to the AXCT2-labeled cells in the thymus. To determine the appropriate tamoxifen dosage to sparsely label cells, we performed a titration experiment with tamoxifen dosages ranging from 0.1-2mg tamoxifen/25g body weight and analyzed after 28 days of tracing. While the higher doses labeled large clusters of cells that likely merged or overlapped (Fig 2A–2E), 0.1mg tamoxifen/25g body weight resulted in sparse labeling suitable for lineage tracing analyses (Fig 2E). Specifically, over a 48 hour trace period, this dosage initiates tracing at a low frequency of 1 in 2884 cells (0.035±0.021%, n = 4)(Fig 2F and 2G).

**Fig 3:**
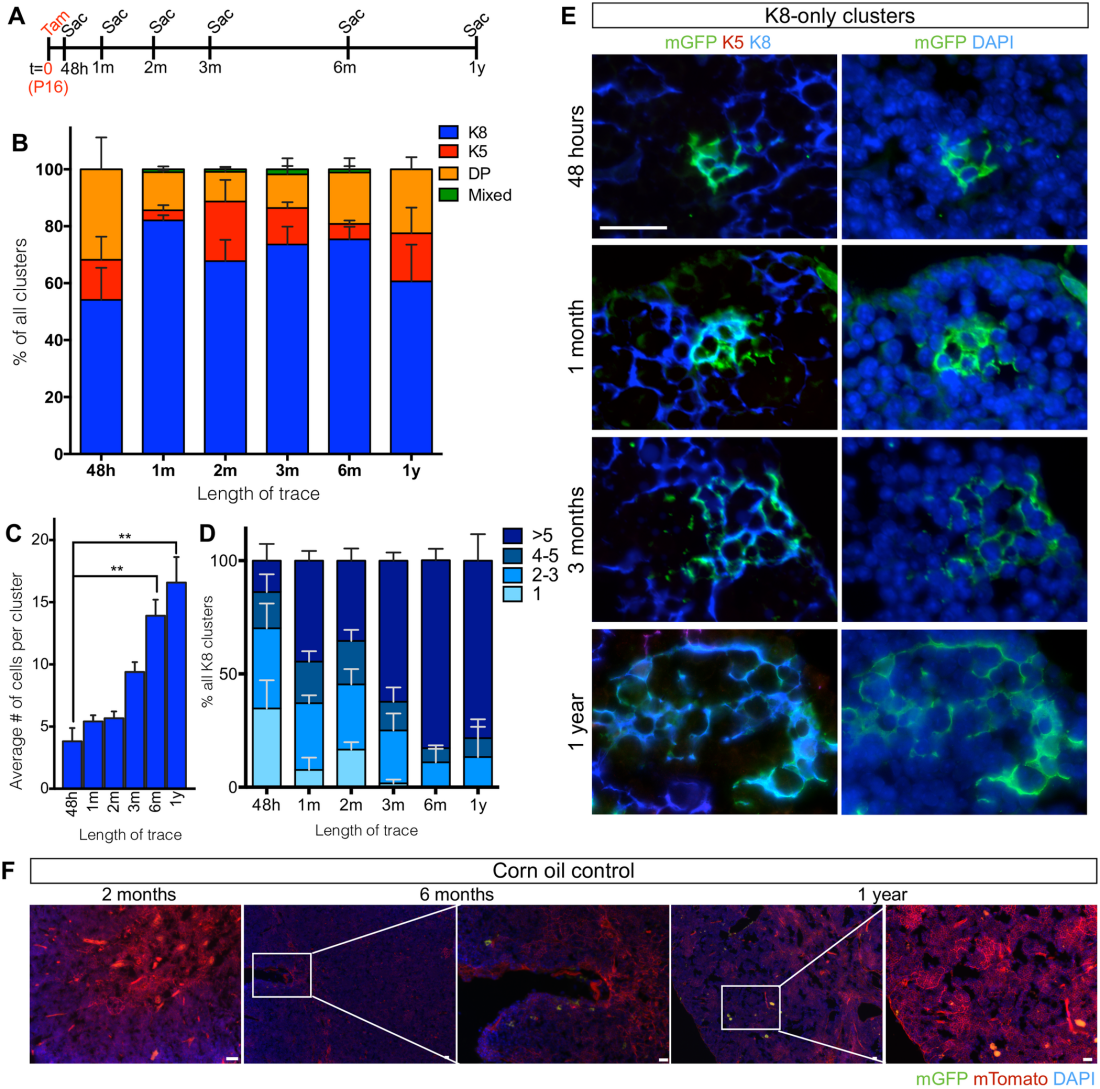
K8-only AXCT2-labeled clusters expand robustly over one year. **(A)** Timeline for AXCT2;mTmG lineage tracing experiments. n = 3 or 4 mice per time point. **(B)** AXCT2-labeled clusters are grouped into four categories according to their K8 and K5 expression—K8 only (blue), K5 only (red), K5 K8 double positive (DP, orange) only and mixed (cluster with more than one type of cells, green). The graph shows the distribution of each cluster type category among all the labeled clusters observed per time point. **(C)** The average number of cells per cluster is plotted for K8-only cluster type at each time point. The differences in average cluster sizes between 48-hour trace and 6 month or 1 year trace are tested for significance. **(D)** The size distribution of K8-only clusters at each time point. **(E)** Representative images of mGFP-labeled K8-only clusters observed at 48 hours, 1 month, 3 months and 1 year after trace initiation. The left panels show co-staining with mGFP, K5 and the K8, and the right panels show the same clusters with DAPI. **(F)** AXCT2;mTmG mice were injected with corn oil at P16, and traced for two months, six months, and 1 year. Ectopic activation of AXCT2 in the AXCT2;mTmG thymus is absent throughout the period of trace. The mTomato signal is expressed constitutively by every cell in the absence of recombination. Graphs depict averages ± SEM, and two-tailed unpaired Student’s-t test was performed to obtain p values of significance. ns—not significant, ** p<0.01. Scale bars represent 20μm.

**Fig 4:**
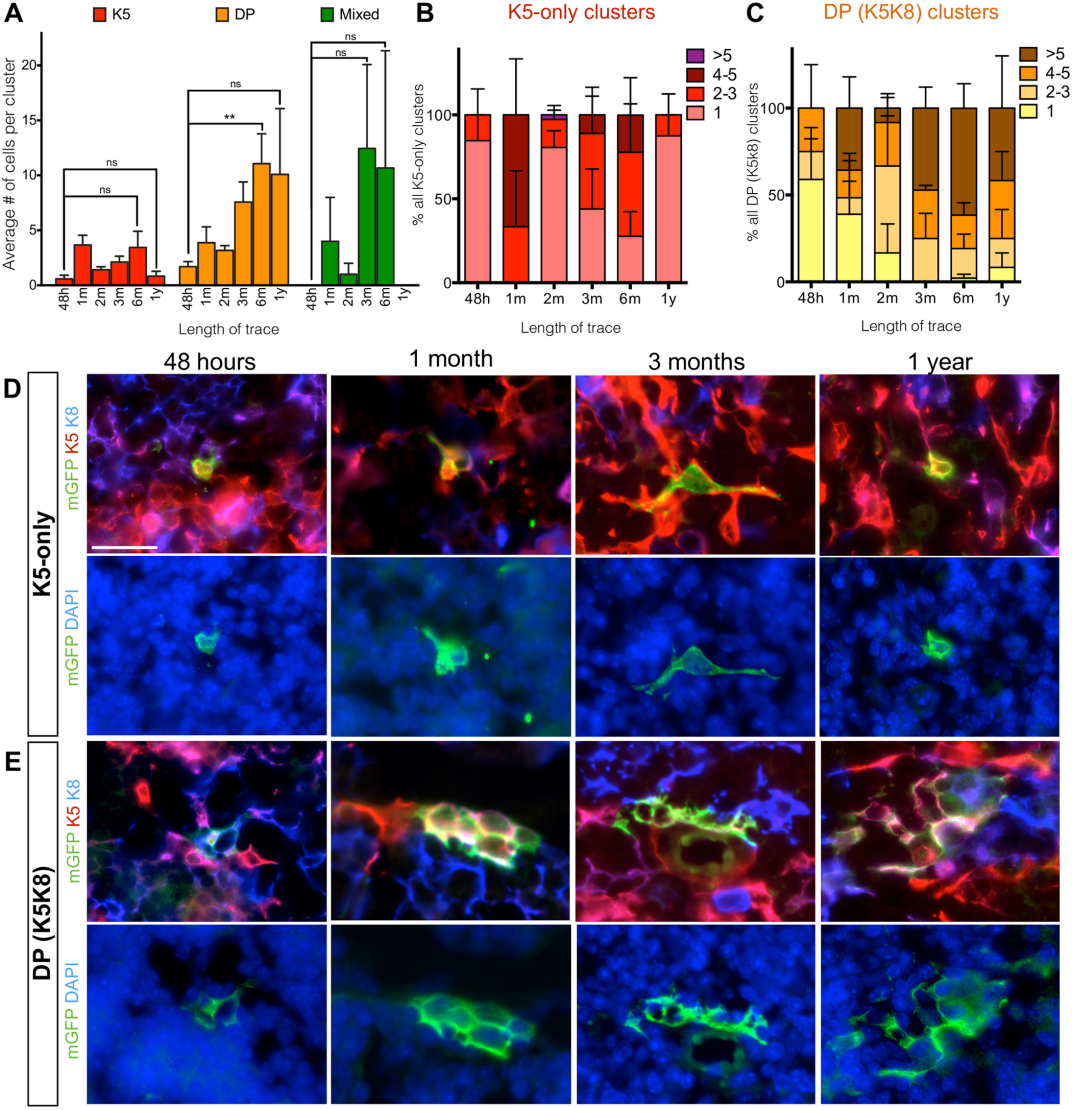
K5-only and DP-only AXCT2-labeled clusters do not expand significantly over time. **(A)** The average number of cells per cluster is plotted for K5-only (red), DP-only (yellow) and mixed (green) cluster types at each time point. The differences in average cluster sizes between 48 hour trace and 6 month or 1 year trace are tested for significance. **(B and C)** The size distribution of K5-only clusters (B) and DP-only clusters (C) at each time point. **(D and E)** Representative images of mGFP-labeled K5-only clusters (D) and DP-only clusters (E) observed at 48 hours, 1 month, 3 months and 1 year after trace initiation. The left panels show co-staining with mGFP, K5 and the K8, and the right panels show the same clusters with DAPI. Graphs depict averages ± SEM, and two-tailed unpaired Student’s t-test was performed to obtain p values of significance. ns—not significant, ** p<0.01. Scale bars represent 20μm.

For all the time points analyzed (Fig 3A), we categorized the mGFP-labeled clusters into four categories according to their marker expression: K8-only (cTECs), K5-only (mTECs), K5K8 DP, and mixed (a cluster with multiple cell types). Most of the clusters contained only K8+ cells (54.16±11.23%), followed by DP cells (31.8±11.2%), K5+ cells (14.04±8.11%) (n = 4) (Fig 3B). Mixed clusters were rarely observed (0–1.8%). In the 48 hour-traced clusters, the average cluster sizes for K8-only, K5-only, and DP-only clusters were 3.81±1.07, 0.58±0.34 and 1.70±0.46 cells per cluster respectively (Figs 3C and 4A). No mixed clusters were observed at this short trace time (Figs 3B and 4A).

### AXCT2-labeled K8-only clusters expand and persist over a year

Over the course of one year after initiating the lineage tracing, most of the labeled clusters retained their single marker phenotype at all time points (>98%) (Fig 3B). There was no labeling observed in AXCT2;mTmG mice injected with corn oil alone over the course of a year (Fig 3F), indicating that the labeling observed was solely induced by tamoxifen. In view of the 10 to 14-day turnover rate of TECs [27], these data suggest that *Axin2*-expresing TECs comprises progenitors that are committed to a single lineage as they give rise to progeny that express the same markers.

Across all time points surveyed, the K8-only clusters exhibited the most consistent increase in cluster size, growing from 3.8±2.1 cells at 48 hours to 16.6±3.6 cells at 1 year, (Fig 3C, p = 0.002, n = 3 to 4 mice per time point). We also tracked the evolution of cluster sizes by their distribution (Fig 3D to 3F). As tracing duration increased, the distribution of the average K8-only cluster size steadily shifted towards larger sizes, and clusters with 1–3 cells became less common. Specifically, the proportion of >5-cell clusters increased from 13.9±7.3% at 48 hours to 78.3±11.7% at 1 year (p = 0.004), and the proportion of clusters with 1–3 cells decreased from 70.1±8.1% at 48 hours to 13.33±13.33% at 1 year (p = 0.01, Fig 3D) (n = 3 to 4 mice per time point).

### No significant expansion of mTEC or DP TEC AXCT2 clusters

In contrast, the average size of a K5-only cluster was less than 4 cells at all time points, and there were no significant differences in cluster sizes amongst the time points surveyed (Fig 4A and 4D). Furthermore, the large majority of the K5-only clusters contained 1–3 cells (ranging from 66.7% to 100% at most time points. Fig 4B). There was no significant difference in the distribution of the 1–3 cell clusters and >5 cell clusters over time (Fig 4B).

While the average size of the DP-only clusters seemingly increased with time, from 1.5±1.7 cells at 48 hours to 10.1±6.0 cells at 1 year (p = 0.2, n = 3 to 4 mice per time point)(Fig 4A and 4E), and the distribution of DP cluster size also tended towards larger sizes (4 or more cells per cluster) at later time points (Fig 4C), the differences were largely statistically insignificant, unlike the K8-only clusters (Fig 3C–3E).

In all, the results from the average cluster size analysis (Figs 3C and 4A) corroborate with those from the distribution analysis (Figs 3D, 4B and 4C) to show that K8-only clusters is the only cluster type that robustly expanded steadily throughout the duration of the lineage trace, as expected of descendants derived from a long-lived progenitor population. Thus, we conclude that the *Axin2*-expressing cTEC population contains long-lived cTEC-lineage specific progenitors that contribute to postnatal development and homeostasis of the thymus.

## Discussion

The development and homeostatic maintenance of many tissues proceeds through multiple types of stem and progenitor cells, which may progressively lead to specialized cells, such as the hematopoietic lineage which consists of progenitors restricted to the lymphoid or myeloid compartments. Our analysis in the thymus demonstrates the existence of a cortical-lineage restricted progenitor, complementing the well-studied medullary lineage progenitor [10,13–16]. Here, we demonstrate the existence of cTEC progenitor in the postnatal thymus through lineage tracing with AXCT2 mice.

### The Wnt target gene *Axin2* labels long-lived cTEC progenitors

When we labeled *Axin2*-expressing cells in AXCT2 mice at P16, we found that K8-only cTEC clusters expanded in a statistically robust manner over time, in contrast to the other subpopulations (Fig 3C to 3E). As the turnover rate of TECs is 10–14 days [27], the lineage tracing data indicate that *Axin2* labels long-lived cTEC progenitors, whose existence was postulated but has yet to be definitively proven [28]. Of note, a short-term cTEC progenitor was identified in the adult thymus through transplantation of EPCAM^+^Ly-51^+^UEA-1^-^PLET1^-^MHCII^lo^ cells with fetal thymus cells in the kidney capsule [11]. It is unclear if this population overlaps with the *Axin2*-expressing population we have identified in our study.

The K8-only AXCT2-labeled clusters are found throughout the thymic cortex, but are more concentrated towards the periphery of the thymus, similar to the pattern observed in the Axin2-LacZ thymus (Fig 1A and 1B). In all, our results demonstrate the existence and behavior of the cTEC progenitor, which contributes to the maintenance of the thymic cortex throughout adulthood. Additionally, since *Axin2* is a Wnt target gene [23], the cTEC progenitors are likely to be active in Wnt/ß-catenin signaling.

The lack of expansion of clusters derived from K5-only and K5K8 DP cells in the AXCT2 mice indicates that *Axin2*-expressing mTECs and DP TECs have no progenitor properties (Fig 4), suggesting that cTEC progenitors are differentially regulated by Wnt signaling.

Interestingly, both the epithelial and hematopoietic compartments of the thymus express Wnt ligands [19,29,30]. Within the epithelial compartment, both the cTECs and mTECs express multiple Wnts [30,31]. Despite the redundancy of Wnt ligand sources in the thymus, abrogating the secretion of Wnt ligands by the TECs resulted in disrupted development of the thymic architecture but not T cell function [31], indicating that Wnt signaling in TECs is dependent on the Wnt ligands secreted by their own population.

### Function and behavior of K5 K8 double positive population are ambiguous

It is commonly thought that the K5K8 DP population contains bipotent progenitors that subsequently gives rise to differentiated mTECs and cTECs [4]. In our lineage tracing study, the majority of the DP clusters observed contained only DP cells, and these DP-only clusters persisted over a year without statistically significant expansion (Fig 4A, 4C and 4E). Mixed clusters were rare throughout all time points of lineage tracing (Fig 3B), suggesting that most of the labeled DP cells did not give rise to K5-only or K8-only TECs. The DP clusters observed in our study are also not restricted to any particular location in the thymus, while the bipotent progenitors are thought to reside at the cortico-medullary junction [4]. Thus, this suggests that the *Axin2*-expressing subset of the postnatal DP population constitutes a distinct TEC subpopulation that is not bipotent *in vivo*, whose exact function remains to be eluded.
